# Susceptibility of AutoML mortality prediction algorithms to model drift caused by the COVID pandemic

**DOI:** 10.1186/s12911-024-02428-z

**Published:** 2024-02-02

**Authors:** Simone Maria Kagerbauer, Bernhard Ulm, Armin Horst Podtschaske, Dimislav Ivanov Andonov, Manfred Blobner, Bettina Jungwirth, Martin Graessner

**Affiliations:** 1 Department of Anaesthesiology and Intensive Care Medicine, School of Medicine, Technical University of Munich, Munich, Germany; 2 Department of Anaesthesiology and Intensive Care Medicine, School of Medicine, University of Ulm, Albert-Einstein-Allee 23, Ulm, 89081 Germany

**Keywords:** Model deterioration, Data shift, Covariate shift, Concept drift, COVID-19, AutoML

## Abstract

**Background:**

Concept drift and covariate shift lead to a degradation of machine learning (ML) models. The objective of our study was to characterize sudden data drift as caused by the COVID pandemic. Furthermore, we investigated the suitability of certain methods in model training to prevent model degradation caused by data drift.

**Methods:**

We trained different ML models with the H2O AutoML method on a dataset comprising 102,666 cases of surgical patients collected in the years 2014–2019 to predict postoperative mortality using preoperatively available data. Models applied were Generalized Linear Model with regularization, Default Random Forest, Gradient Boosting Machine, eXtreme Gradient Boosting, Deep Learning and Stacked Ensembles comprising all base models. Further, we modified the original models by applying three different methods when training on the original pre-pandemic dataset: (1) we weighted older data weaker, (2) used only the most recent data for model training and (3) performed a z-transformation of the numerical input parameters. Afterwards, we tested model performance on a pre-pandemic and an in-pandemic data set not used in the training process, and analysed common features.

**Results:**

The models produced showed excellent areas under receiver-operating characteristic and acceptable precision-recall curves when tested on a dataset from January-March 2020, but significant degradation when tested on a dataset collected in the first wave of the COVID pandemic from April-May 2020. When comparing the probability distributions of the input parameters, significant differences between pre-pandemic and in-pandemic data were found. The endpoint of our models, in-hospital mortality after surgery, did not differ significantly between pre- and in-pandemic data and was about 1% in each case. However, the models varied considerably in the composition of their input parameters. None of our applied modifications prevented a loss of performance, although very different models emerged from it, using a large variety of parameters.

**Conclusions:**

Our results show that none of our tested easy-to-implement measures in model training can prevent deterioration in the case of sudden external events. Therefore, we conclude that, in the presence of concept drift and covariate shift, close monitoring and critical review of model predictions are necessary.

**Supplementary Information:**

The online version contains supplementary material available at 10.1186/s12911-024-02428-z.

## Introduction

When developing machine-learning (ML) models, one should keep in mind that the environment in which they are applied can change with the consequence of a possible deterioration in model performance. Changes usually happen gradually and are summarized under the term “data drift” [1, 2]. The phenomenon of data drift can concern input as well as output parameters. In commercially used models, it is common to perform updates at regular fixed time intervals to counteract these changes [3].

However, the recent COVID pandemic has taught us that environmental changes can also occur abruptly with unpredictable consequences for ML models. This was felt in the free economy, where customer behaviour changed due to the lockdown, forcing companies to adjust their sales models [4, 5].

In the medical field, COVID-related changes were particularly noticeable; they affected not only intensive care and infectiology but also significantly impacted routine care at our hospitals [6]. With the beginning of the pandemic, elective procedures were postponed, and there were fewer hospital admissions overall, while the case-mix index, length of hospital stay and mortality rates increased [7].

These changes, which occurred so unpredictably, also led to an impairment of machine learning models in the medical field. For example, COVID-induced performance drift has been shown for mortality and sepsis prediction models [1, 8].

It is not possible to predict how such sudden changes in the characteristics of the patient collective or the surgical spectrum will affect ML algorithms.

For intended use in clinical routine, however, it is of enormous importance that ML models function reliably and display a certain robustness. Otherwise, elaborate adaptation measures after a sudden event would lead to significant system downtime and restrict the use of an algorithm in practice.

In addition, changes in external circumstances are not always as obvious as in the case of the COVID pandemic and may only be noticed with some delay. Therefore, the question arises whether it is possible to increase the robustness of machine learning models from the outset.

In the present study, we addressed this question by examining how model performance is affected by the COVID pandemic using the example of models created with AutoML for the prediction of postoperative mortality from preoperatively available data. We analysed for changes in the most important input parameters and tried to prevent the decline in predictive performance in advance by taking measures during model development.

## Patients and methods

### Study design

The study was approved by the Ethics Committee of the Technical University of Munich (TUM), School of Medicine (253/19 S-SR, approval date 11/06/2019) and registered in Clinical Trials (NCT04092933, initial release 17/09/2019). Informed consent was waived because of the retrospective study design. We conducted the study in concordance with the TRIPOD guidelines for reporting predictive model studies.

**Fig. 1:**
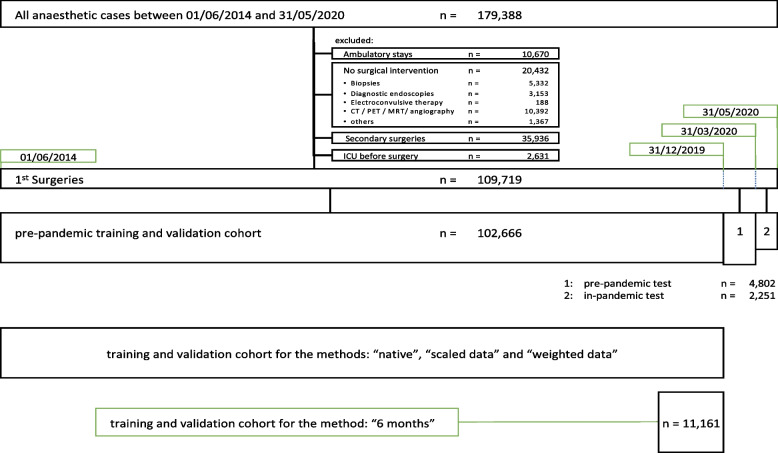
Strobe Diagram.  Applying exclusion criteria as shown, 109,719 anaesthetic cases remained in the whole dataset. Training and validation collective for the “native” as well as the “scaled” and “weighted” models consisted of 102,666 cases. The “6 months” method dataset comprised 11,161 cases. CT computed tomography, PET positron emission tomography, MRT magnetic resonance tomography, ICU intensive care unit

We included data from all patients undergoing non-cardiac surgery at a single German University Hospital between June 2014 and May 2020. Only the first surgery of each patient during one hospital stay was included, subsequent surgeries were disregarded. If the patient was readmitted due to another disease at a later time, this was treated as a separate case in the dataset. A patient did not appear as a separate case if (i) he or she was readmitted with the same diagnosis within 30 days, (ii) if it was a planned readmission according to a treatment plan, (iii) treatment was interrupted for less than 24 h, or (iiii) returned from a rehabilitation facility. We included patients of all age groups and elective as well as urgent procedures. Patient numbers and exclusion criteria are depicted in Fig. 1.

Model training and validation were performed with the dataset ranging from June 2014 to December 2019. Models aimed to predict in-hospital mortality after a surgical procedure. Performance metrics were determined using two testing cohorts with data that was not included in the training or validation data set: The first testing cohort comprised patients undergoing surgery from January to March 2020 (pre-pandemic testing cohort), the second testing cohort consisted of patients treated in April and May 2020 during the first pandemic wave (in-pandemic testing cohort). All metrics presented in this study refer to these two sets of test data. The designated endpoint of the models was postoperative in-hospital mortality. The prediction is based on preoperatively available data from the anaesthesiologic patient data management system (PDMS, brand: QCare, manufacturer: HIM-Health Information Management GmbH, Bad Homburg, Germany) which was used by the physician to conduct the pre-anaesthesia visit and to assess patient history including pre-existing illnesses and medication. Patient core data was derived from the hospital information system (SAP i.s.h.-med) and preoperatively available laboratory values from the laboratory information system (swisslab Lauris). Thus, all preoperatively available information on patients’ individual data, medication, and laboratory values, as well as all codes for the type of surgical intervention, were offered as input parameters. Since the collection or non-collection of data in the pre-operative course could also contain hidden information, we created a dichotomous variable about its presence for each variable with missing values and included it in the model.

Further, we did not perform any imputation as part of data pre-processing. Instead, we used AutoML’s internal algorithm, which replaces missing values of continuous variables with the mean and for categorical variables with the modal value (https://docs.h2o.ai/h2o/latest-stable/h2o-docs/data-science/algo-params/missing_values_handling.html) [9]. Data extraction from the clinical systems and processing was performed as described before [10, 11]. In short, we removed redundant data documented in the clinical systems more than once. Numerical and categorical data were transferred unchanged, and other data types had to be modified: Free text was subjected to a quantity-based search for keywords, which were then used as categorical input parameters. Drugs were grouped according to the first four digits of their Anatomical Therapeutic Chemical (ATC) code, and the extremely fine-grained codes of the German OPS system [12] for classifying medical interventions were summarised. In the end, of about 12,000 parameters from the raw data, 2,775 possible input variables remained for modelling.

### Model development

Models were developed using the H2O framework in R (version 4.3.2). We applied the h2o.automl() function. The model types used were a Generalized Linear Model with regularization (GLM), Default Random Forest (DRF), Gradient Boosting Machine (GBM), eXtreme Gradient Boosting (XGBoost), a fully-connected multilayer neural network (Deep Learning), and Stacked Ensembles including ensembles of all base models (“all models”) and the best model of each algorithm family (“best of family”) [13]. As GLM, a binomial regression model was used with ridge regularization according to the default settings. (https://docs.h2o.ai/h2o/latest-stable/h2o-docs/automl.html) [9]. We configured AutoML to run for a specific amount of time. Once this time limit is reached, the process stops, regardless of the number of models that have been trained. Using a total computing time of 125 h, 649 models in total were created with different random hyperparameters: 269 native models and 62, 46 and 272 with the three different adaptation methods. The whole dataset ranging between June 2014 and December 2019, serving as a training and validation dataset, was split once using a stratified 80:20 split so that there were approximately equal proportions of deaths in each of the two sets. We used the pre-pandemic data from January to March 2020 and in-pandemic data from April and May 2020 as test sets. The oversampling process included in the AutoML framework (“balance_classes”-parameter) was used to counteract the class imbalance [9]. Individual model creation was stopped when the area under precision recall curve (AUPR) did not increase for 100 rounds. We chose to optimise using the AUPR [14], because poor precision-recall trade-off was a weakness of the models we created in former studies and is a known limitation of AutoML models [11, 15].

Variable importance was determined using the h2o.permutation.importance() function for the Stacked Ensemble models and the h2o.varimp() functions for the other models (https://docs.h2o.ai/h2o/latest-stable/h2o-docs/variable-importance.html) [9].

Calculations were performed on the Linux Cluster of Leibniz Supercomputing Centre in Munich on 20 cores Intel Xeon-CPU with 800 GB of RAM and a peak performance of 1400 TFlop/s.

### Methods to improve model robustness

After training the models on the original dataset comprising data between June 2014 and December 2019, we tried to increase the robustness of these native models with three different measures, namely, first, weighting the input features with a factor as follows (method: “weight”): The training and validation data set comprised a total of 2040 days. Here, the first day with available data in the dataset was assigned the weight $\frac{1}{2040}$, and the parameter values of the last included day in the dataset used for training and validation, 31/12/2019 (day 2040), were weighted with the factor 1. The other days were weighted with a quotient of the day n and the number of total days ($\frac{n}{2040}$) in the data set. The models were then trained with the weighted features.

Second, only the data from June - December 2019 were used as the training and validation dataset to use the most recent data possible (method:”6 months”). This method applies a binary step function where data before the cut-off date is weighted with 0 and from the defined cut-off point on with 1.

Third, the numerical input parameter values were subjected to a z-transformation (*z =* $\frac{x-\mu }{\sigma }$, where x is the respective value, µ the mean and σ the standard deviation of the sample). This method is referred to below as “scaled”. There are two approaches to carrying out this scaling: Firstly, the mean value and standard deviation of the entire data set can be determined, and these scaled values can be used as input. On the other hand, the training and validation set can be scaled together for model development, and then the pre- and in-pandemic data set can be scaled separately. The second method may allow a better adjustment to the covariate shift, but it assumes that data from the pandemic already exists, so it cannot be used pre-emptively.

### Statistical analysis

Analysis was performed using R (Version 4.3.2, R Foundation for Statistical Computing, Vienna, Austria). Comparison metrics for the models were their area under the receiver operating characteristic (AUROC) and area under the precision-recall curve (AUPR), shown with their 95%-confidence interval. Kolmogorov-Smirnov-Test was performed to detect differences in the probability distribution of the input features. Model performance before and in the pandemic was compared by paired t-test. Performances of the native models compared with model performance after the corrective actions “weight”, “6 months”, and “scaled” were quantified by means of one-way ANOVA with post-hoc Tukey HSD test.

## Results

### Native model performance

**Fig. 2:**
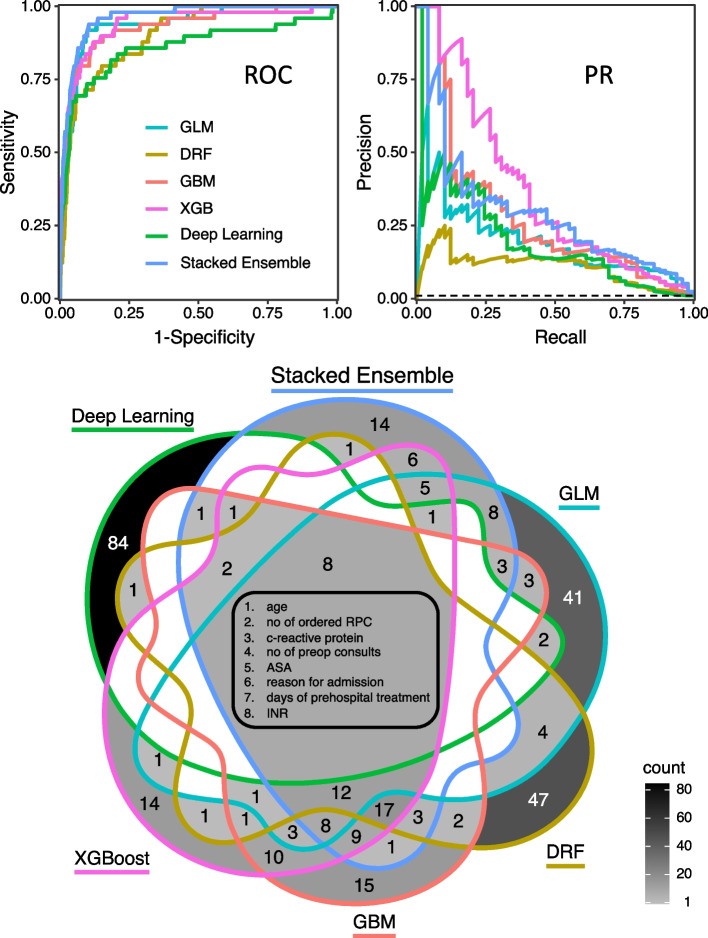
ROC- curves (top left), PR-curves (top right) and Venn-Diagram (bottom) of the best pre-pandemic model of each family. The dashed line in the precision-recall plot showed the baseline which is the precision that would be achieved if the model always predicted the negative class. The Venn Diagram includes the 100 most important features of the best model of each family. Intersections without numbers are empty sets, the outer areas (84,14,41,47,15, and 14 features respectively) occur only in the top 100 of the respective model. The centre of the diagram shows the 8 parameters that occur in each of the models. GLM: Generalized Linear Model, DRF: Default Random Forest, GBM: Gradient Boosting Machine, XGB: eXtreme Gradient Boosting, RPC: red packed cells, ASA: American Society of Anesthesiologists, INR: International Normalized Ratio

 In total, 269 native models were developed on the dataset ranging from June 2014 to December 2019. These models showed excellent AUROCs and acceptable AUPRs on the pre-pandemic test set. Stacked Ensemble models performed best with a mean AUROC of 0.95 and a mean AUPR of 0.26. The ROC- and PR-curves of the best model of the respective family in the pre-pandemic test set are depicted in Fig. 2. Applying the best pre-pandemic models to the in-pandemic test set, mean model evaluation metrics AUROC and AUPR declined significantly in GBM, XGBoost, Deep Learning and Stacked Ensemble models (Table 1). As an additional information we present F1 scores of the native models pre- and in pandemic in Supplemental Figure S1.

**Table 1:** Pre- and in-pandemic performance of the native models.This table shows mean values and standard deviations of pre- and in-pandemic areas under receiver operating characteristic curves (AUROC) and areas under precision-recall curves (AUPR) as well as their percentage changes

		AUROC					AUPR				
model	n	validation set	pre-pandemic	in-pandemic	% Change	*p*-value	validation set	pre-pandemic	in-pandemic	% Change	*p*-value
GLM	1	0.93	0.94	0.90	-3.85		0.17	0.19	0.08	-57.65	
DRF	2	0.85 (0.01)	0.90 (0.00)	0.85 (0.02)	-4.98 (1.59)	0.14	0.07 (0.00)	0.09 (0.02)	0.07 (0.00)	-26.26 (15.57)	0.3
GBM	32	0.86 (0.05)	0.86 (0.06)	0.81 (0.06)	-6.26 (5.17)	< 0.001	0.10 (0.02)	0.19 (0.04)	0.07 (0.03)	-59.95 (18.33)	< 0.001
XGBoost	198	0.90 (0.01)	0.93 (0.01)	0.88 (0.02)	-5.36 (1.87)	< 0.001	0.11 (0.02)	0.24 (0.04)	0.07 (0.01)	-72.08 (8.10)	< 0.001
Deep Learning	22	0.88 (0.03)	0.90 (0.02)	0.84 (0.04)	-6.30 (3.15)	< 0.001	0.10 (0.02)	0.14 (0.03)	0.05 (0.01)	-62.17 (7.57)	< 0.001
Stacked	14	0.95 (0.01)	0.95 (0.01)	0.91 (0.01)	-4.70 (0.73)	< 0.001	0.27 (0.21)	0.26 (0.03)	0.09 (0.00)	-65.91 (6.86)	< 0.001
all	269	0.90 (0.03)	0.92 (0.03)	0.87 (0.04)	-5.50 (2.58)	< 0.001	0.12 (0.06)	0.22 (0.05)	0.07 (0.02)	-69.11 (11.41)	< 0.001

*GLM* Generalized linear model, *DRF* Default random forest, *GBM* Gradient Boosting Machine, *XGBoost* eXtreme Gradient Boosting, *Stacked* Stacked Ensemble, *all* mean values of all models, *n* number of models created by AutoML. Pre- and in-pandemic AUROC and AUPRC are compared using paired t-test where p<0.05 was considered statistically significant. % Change indicates the mean percentage change of each model family

### Features of the native models and probability distributions

About 12,000 possible input features from the raw data were available for model development, with 2,775 being used for model creation. The most important common feature in the best pre-pandemic native model of each family was age; the other features that were commonly used in the best native models were number of preoperatively ordered red blood cell concentrates, c-reactive protein, number of preoperatively requested consults, ASA score, reason for admission, number of prehospital treatment days and international normalized ratio.

During the pandemic, there was a change in the probability distribution of input features. The Kolmogorov-Smirnov test performed on the validation set and the in-pandemic test set revealed significant differences on a large number of features including the common ones of the best pre-pandemic models.

The probability of occurrence of the endpoint, post-operative in-hospital mortality, did not change significantly during the pandemic. Patient-characterizing features and their percentage change during the pandemic are shown in Table 2.

**Table 2:** Changes in feature distribution and Kolmogorov-Smirnov-Test results of the most important cohort-describing pre-pandemic features

	pre pandemic validation *n* = 102,666		pre pandemic test *n* = 4,802		KS-test^§^ *p*-value	in pandemic *n* = 2,251		KS-test^#^ *p*-value
**Deceased**	834	(0.8%)	49	(1.0%)	1	22	(1.0%)	0.458
**Age**^a^	56	(38, 70)	56	(38, 70)	0.292	58	(40, 72)	< 0.001
**Sex**					1			0.720
Male	55,640	(54.2%)	2,546	(53.0%)		1,186	(52.7%)	
Female	47,026	(45.8%)	2,256	(47.0%)		1,065	(47.3%)	
**BMI missing**	33,445	(32.6%)	831	(17.3%)	0.711	484	(21.5%)	< 0.001
**BMI**	25.2	(22.5, 28.6)	25.2	(22.5, 28.7)	0.914	24.9	(22.2, 28.4)	0.034
**Lab request RPC’s ordered**	27,727	(27.0%)	1,485	(30.9%)	1	849	(37.7%)	< 0.001
**No of ordered RPC’s**^a^	4.00	(2.00, 4.00)	4.00	(2.00, 4.00)	1	4.00	(2.00, 4.00)	< 0.001
**Laboratory CRP missing**	47,588	(46.4%)	1,878	(39.1%)	1	879	(39.0%)	< 0.001
**Laboratory CRP**^a^	0.30	(0.10, 1.00)	0.30	(0.10, 1.10)	0.558	0.30	(0.10, 1.60)	< 0.001
**Number of consults before surgery**^a^	2.00	(1.00, 3.00)	2.00	(1.00, 4.00)	0.211	3.00	(2.00, 4.00)	< 0.001
**ASA missing**	30,624	(29.8%)	783	(16.3%)	0.999	440	(19.5%)	< 0.001
**ASA**^a^					1.000			< 0.001
I	20,202	(28.0%)	1,002	(24.9%)		415	(22.9%)	
II	37,049	(51.4%)	1,950	(48.5%)		889	(49.1%)	
III	14,102	(19.6%)	1,011	(25.2%)		474	(26.2%)	
IV	642	(0.9%)	53	(1.3%)		33	(1.8%)	
V	47	(0.1%)	3	(0.1%)		0	(0.0%)	
**Reason for admission**^a^					0.513			< 0.001
Obstetric	20,369	(19.8%)	911	(19.0%)		553	(24.6%)	
Normal case	56,961	(55.5%)	2,935	(61.1%)		1,233	(54.8%)	
Organ donor	21,239	(20.7%)	753	(15.7%)		359	(15.9%)	
Accident	4,001	(3.9%)	197	(4.1%)		106	(4.7%)	
Full inpatient with pre-inpatient treatment	96	(0.1%)	6	(0.1%)		0	(0.0%)	
**Treatment days pre-inpatient**^a^	1.00	(0.00, 1.00)	1.00	(0.00, 2.00)	0.204	1.00	(0.00, 1.00)	< 0.001
**Laboratory INR missing**	24,796	(24.2%)	647	(13.5%)	1	283	(12.6%)	< 0.001
**Laboratory INR**^a^	1.00	(0.90, 1.00)	0.90	(0.90, 1.00)	0.902	0.90	(0.90, 1.00)	< 0.001

The Kolmogorov-Smirnov-Test was performed between the pre-pandemic validation and the pre-pandemic test set(§), and the pre-pandemic validation and the in-pandemic test set(#), a p-value < 0.05 indicates that the distributions of the respective parameter differ significantly from each other

The table shows the common parameters of the best pre-pandemic model of each family which are marked with ^a^. Sex and body mass index (BMI) were included in the table to better describe the patient collective. The number and percentage of missing values are referred to as "missing" and are also listed in the table. The absolute number and the percentage share are shown for discrete variables and the median and interquartile range for continuous variables in brackets

*BMI* Body mass index, *RPC’s* Red packed cells, *CRP* c-reactive protein, *ASA* American Society of Anesthesiologists physical score [16], *INR* International normalized ratio

### Attempts to increase robustness

None of the modifications carried out could prevent the significant deterioration in performance. Occasionally, individual models did not drop quite as much in the pandemic or even improved, but the performance metrics of these models did mostly not perform well on the pre-pandemic test data set.

In detail, the weighting method, by which 62 models were created, led to a highly significant drop in AUROC of the Deep Learning models accompanied by a decline in precision-recall trade-off in the pre-pandemic test set which remained almost unchanged in-pandemic. The performance of the other model families remained mostly unaffected before the pandemic but also suffered from performance loss during the pandemic.

Training the models with data obtained no longer than 6 months before the pandemic led to a slightly worse pre-pandemic performance according to AUROC and to a deterioration of pre-pandemic precision-recall trade-off in most of the 272 models. Furthermore, the use of the most recent data could also not prevent the decline during the pandemic. It is noticeable that the Deep Learning and GBM models showed a large range regarding AUROC.

The scaling method with scaling performed on the whole dataset yielded 46 models. Applying this method, the individual models no longer showed such a large fluctuation in performance metrics within their families, although the drop in performance could not be prevented here either. In the main manuscript we refer to the method “scaling” as scaling on the whole dataset as described above. Results of separately performed scaling for training/validation and pre-and in-pandemic test data are presented in Supplemental Figure S2. Here, too, a deterioration in performance was observed during the pandemic.

**Fig. 3:**
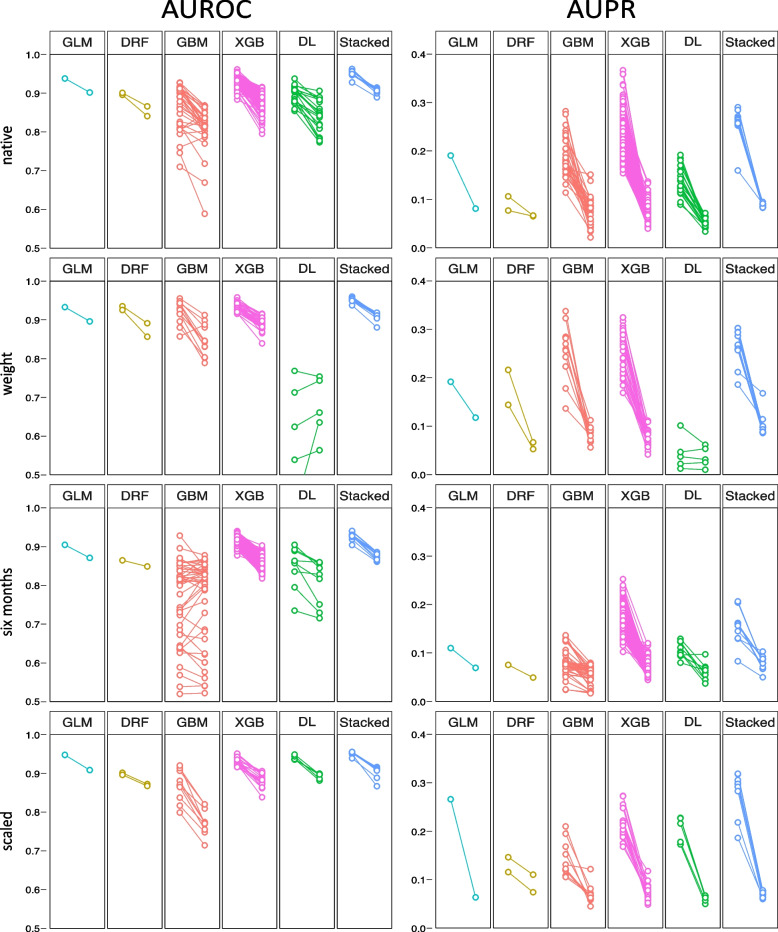
AUROC and AUPR pre- and in-pandemic. Two points that belong together are each connected by a line. The respective left point represents model performance on the pre-pandemic test set, the right point on the in-pandemic test set. Changes in area under receiver-operating characteristic (AUROC) curve are depicted in the left column, changes in area under precision-recall (AUPR) curve are depicted in the right column. This graphic shows all the created models of each family. Most models suffer from a performance loss in the pandemic, not only the native ones, but also those modified by means of the “weight”, “6 months” and “scaled” methods. GLM: Generalised Linear Model, DRF: Default Random Forest, GBM: Gradient Boosting Machine, XGB: eXtreme Gradient Boosting, DL: Deep Learning, Stacked: Stacked Ensemble

Loss of model performance according to AUROC and AUPR in all applied methods is shown in Fig. 3. A comparison of pre- and in-pandemic evaluation metrics according to model families and modification methods can be found in Supplemental Tables S1 and S2.

### Common features after model modification

We analysed the 100 most important features of the best model of each family and determined their common intersection. Applying the “weighted data” method, of the top 100 features used in the best pre-pandemic model of each family, only 1 feature was common in all models, namely “age”, which is the most important mortality-determining factor overall.

Using the “6 months” method, the best models of each family had 13 features in common with age, reason for admission and number of preoperative consults being the most important.

The “scaled” models had 5 factors in common, including age, and several laboratory values.

**Fig. 4:**
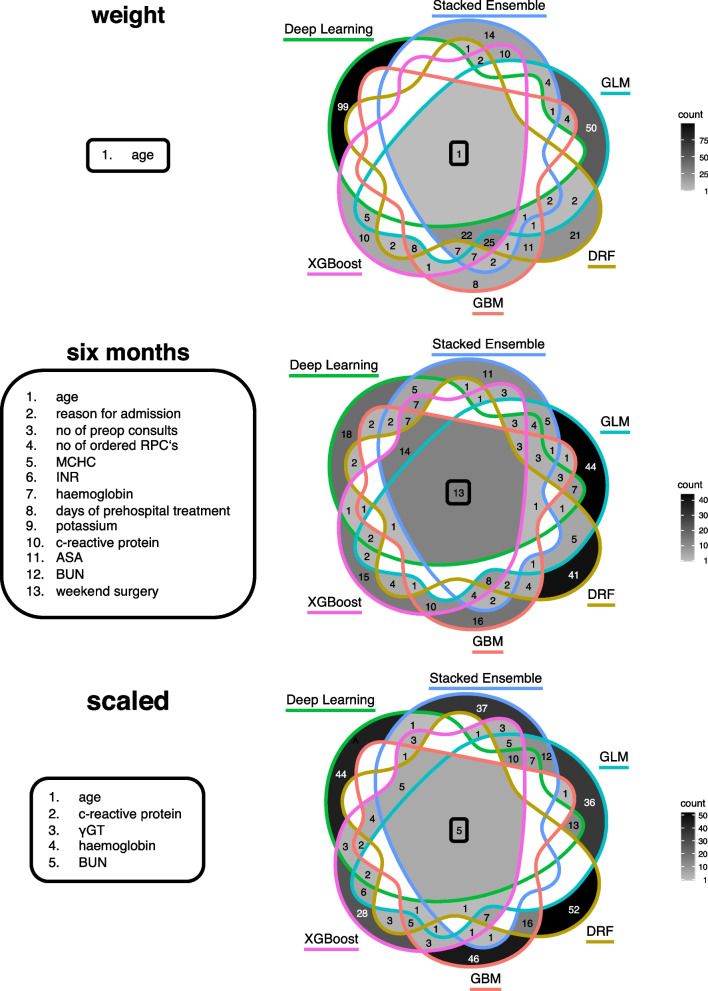
Venn diagrams after model modification. Diagrams show the 100 most important features of the best pre-pandemic model of each family after the methods “weight”, “6 months” and “scaled” were applied. The centre of each diagram shows the number of parameters that occur in each of the models, with “age” being the number one common feature in each of the methods. The text boxes on the left indicate which parameters are involved in all models sorted by their importance. RPC’s: red packed cells, INR: international normalized ratio, γGT: gamma-glutamyl transferase, ASA: American Society of Anaesthesiologists Physical Score [16], BUN: blood urea nitrogen, MCHC: mean corpuscular haemoglobin

Venn diagrams of the top 100 factors of the best pre-pandemic model of each family are shown in Fig. 4.

### Feature importance pre- and in-pandemic

Models that performed best in the pandemic mostly used different variables or the variables used differed in their importance compared with models of the same family that performed best before the pandemic. An overview of variable importance in the models that performed best pre-pandemic and in-pandemic is provided in the appendix (Supplemental Table S3).

## Discussion

In our study, we used the AutoML framework which is becoming increasingly popular due to its efficiency in creating models with little expenditure of time. Although there is not much data on this topic, it is assumed that AutoML is susceptible to data shifts [15]. We can confirm this assumption in our study, as all parametric and non-parametric methods provided by this framework showed significant deterioration when exposed to COVID-related changes.

Taken together, a loss in performance of AutoML models for the prediction of postoperative mortality due to the COVID pandemic was clearly recognizable and evident in the precision-recall trade-off as well as in the AUROC. Weighing the input features, using only the most recent data or scaling the input features did not have an impact on the robustness of the models.

In creating the models, we applied a data set with a great number of features. Although we removed redundant variables during preprocessing, we intentionally left highly correlated variables in the final data set (for example, haemoglobin and haematocrit or liver and kidney laboratory panels) to avoid loss of information. As a result, importance was split across several variables and was often relatively low in the individual case. Despite the high number of input features, there were relatively few overlaps in the common parameters of the best models. Despite this diversity, the vast majority of these models deteriorated when using data from the first pandemic wave. This is a sign that the pandemic was a sudden multifactorial event, especially in its initial phase. We know from the recent medical literature that conditions in our hospitals became different with the onset of the pandemic [7, 17] including a change in demographics and the types of surgeries performed [18, 19] which could also be observed in our patient collective.

The fact that the COVID-pandemic caused a data shift that exerts a negative influence on the performance of machine learning algorithms being trained with historical data has already been demonstrated in the field of medicine [1, 8, 20]. However, it can only be speculated what consequences this will have in practice and what measures should be taken.

It can be observed in many models that AUROCs decline but remain in an acceptable range, whereas the precision-recall-trade-off decreases and, due to a low baseline of about 1%, is better than random guessing but may not be satisfactory in practice [21]. In the clinical setting, the performance of a binary classification model depends on the threshold settings which are adapted to a specific clinical situation. General assumptions can be made regarding AUROC and AUPR: While the high AUROC means that a model is generally good at discriminating between classes, the low AUPR indicates that it is not effective at identifying true positive cases, which would usually limit its use in clinical practice. Especially in times of limited resources, this suboptimal trade-off can lead to false alarms or unnecessary interventions. This limitation is exacerbated in the presence of a covariate shift, as occurred at the beginning of the COVID pandemic. Consequently, usage of these models could, for example, lead to a rising number of patients admitted to high-dependency units as a consequence of an incorrectly assumed high risk of potentially fatal complications. Such a misjudgement would place additional burden on an already battered healthcare system.

The domain of medicine is known as a so-called non-stationary environment where several types of data drift can happen either gradually or abruptly [22]. Various types of such data shifts are described in the literature, including concept drift [23], prior probability shift, posterior probability shift and covariate shift [24, 25]. Concept drift means that the relationship between input variables and output changes. One indication of concept drift in our study is the fact that the models that performed best before the pandemic and those that performed best in the pandemic mostly use different input variables. For example, the deep learning model that works best with the pre-pandemic dataset is primarily based on surgical codes, while the one that provides the best prediction in-pandemic relies on other parameters such as consults and laboratory values.

“Prior probability shift” happens due to a change in the initial probabilities of events or outcome categories. This means that the input variables remain the same, but the distribution of the output variable changes. A “posterior probability shift” accounts for the change in probabilities of events after the consideration of new data. Both phenomena are rather out of the question for our data set as the mortality rate remains unchanged in the whole time period.

The changes that lead to a deterioration of the models in our case are most likely to be a covariate shift. The term “covariate shift” refers to a change in the probability distribution of the input data while the conditions or events remain the same [26]. This problem can occur when the model is applied under different circumstances than those under which it was trained. In our case, this fact is illustrated by significant Kolmogorov-Smirnov-test findings between pre-pandemic data and the in-pandemic test dataset.

The phenomenon of covariate shift and concept drift occurs in most real-world data sets [26]. Many authors therefore also investigated methods aimed at compensating for this shift as well as methods for detecting the drift [24, 27–30]. To achieve balanced covariates, for example, it is possible to use propensity score matching [31] or to perform re-training with or without model weighting [23].

Unfortunately, many of the proposed compensation measures require real-world post-intervention data [29] which means that, in the case of sudden events like the COVID pandemic, it would be necessary to retrain the model with new data or to anticipate a change in the parameter distribution. Therefore, it is necessary to review the real-world, clinical and demographic data of the patient population regularly. This is critical to ensure that the machine learning model continues to respond to current and representative data and thus maintains its accuracy and relevance.

In order to be able to do without such re-training, it would be better to develop models that are more robust against sudden changes from the outset. In our study, we address this question by applying several strategies before training the original model. One simple adjustment we tried was assigning different weights to the features in such a way that the older the data, the weaker they are weighted. To further increase this measure, in the next step we only took data from the last 6 months of the training period to build the model. The fact that these two methods could not prevent model degradation is just another indication that the data drift to which the model was subjected did not happen gradually, but suddenly. Our third approach, the concept of normalization of data, is a common practice in the field of machine learning. It is believed that many algorithms work better when all features have the same scale and are centred around the zero point [32]. However, even this approach did not show any significant effects on model degradation.

From the present results, it is extremely important to critically evaluate model predictions in changing concomitant circumstances. Further studies are needed to elucidate the best methods of model surveillance and adaptation to achieve reliable predictions especially when environmental changes do not only occur gradually but suddenly.

### Strengths and limitations

Our models were created using data from only a single centre in Germany which is probably why many national and local circumstances are reflected in the data set used. The impact and directives at the beginning of the COVID pandemic also differed from country to country, reflecting German particularities here. However, with a total of over 109,000 cases, we used a representative dataset from a university hospital and assumed that COVID-related cuts have led to significant changes in the clinical setting in almost every country.

The use of domain adaptation methods could be useful in this context [33, 34]. Still, it should be borne in mind that at the beginning of the pandemic, it was not foreseeable to what extent the data distribution would change. Therefore, such an adjustment could only be made retrospectively.

The aim of our work was a general comparison of the models that can be created in AutoML. We are aware that different thresholds would be chosen for different clinical applications, which is why we preferred AUROC and AUPR over metrics that require the specification of a correct threshold.

Finally, there are a number of other modifications that could be applied, such as further non-linear methods of weighting data. However, we have deliberately chosen simple mathematical and data set adaptations to the models in order to make them comprehensible to clinicians and applicable in practice.

## Conclusions

Both the parametric and non-parametric methods represented in the AutoML framework experienced a loss of performance at the beginning of the COVID-pandemic which was caused by concept drift and covariate shift. Simple adjustments in model building like weighing, scaling or using only the most recent data did not make them more robust. Therefore, if models are intended for use in clinical routine, model surveillance plays a crucial role in detecting and reacting to changes early on.

### Supplementary Information

**Additional file 1.**

## Data Availability

The dataset analyzed during this study is not publicly available due to legal regulations. To gain access, proposals should be directed to the corresponding author. Requestors will need to sign a data access agreement. The underlying code for this study is available in GitHub and can be accessed via this link: https://github.com/BernhardUlm/COVIDMortality.
