# Correction: Susceptibility of AutoML mortality prediction algorithms to model drift caused by the COVID pandemic

**DOI:** 10.1186/s12911-024-02454-x

**Published:** 2024-02-19

**Authors:** Simone Maria Kagerbauer, Bernhard Ulm, Armin Horst Podtschaske, Dimislav Ivanov Andonov, Manfred Blobner, Bettina Jungwirth, Martin Graessner

**Affiliations:** 1 Department of Anaesthesiology and Intensive Care Medicine, School of Medicine, Technical University of Munich, Munich, Germany; 2 Department of Anaesthesiology and Intensive Care Medicine, School of Medicine, University of Ulm, Albert-Einstein-Allee 23, 89081 Ulm, Germany

**Correction: Kagerbauer et al. BMC Medical Informatics and Decision Making (2024) 24:34**

10.1186/s12911-024-02428-z

Following the publication of the original article [1], the authors reported typesetting errors and a typo.

The first typesetting error was found in the Methods’ section of the Abstract. The numbering in the section was mistakenly linked to the reference, as follows:

We trained different ML models with the H2O AutoML method on a dataset comprising 102,666 cases of surgical patients collected in the years 2014–2019 to predict postoperative mortality using preoperatively available data. Models applied were Generalized Linear Model with regularization, Default Random Forest, Gradient Boosting Machine, eXtreme Gradient Boosting, Deep Learning and Stacked Ensembles comprising all base models. Further, we modified the original models by applying three different methods when training on the original pre-pandemic dataset: (Rahmani K, et al., Int J Med Inform 173:104930, 2023) we weighted older data weaker, (Morger A, et al., Sci Rep 12:7244, 2022) used only the most recent data for model training and (Dilmegani C, 2023) performed a z-transformation of the numerical input parameters. Afterwards, we tested model performance on a pre-pandemic and an in-pandemic data set not used in the training process, and analysed common features.

The correct sentence should have been:

We trained different ML models with the H2O AutoML method on a dataset comprising 102,666 cases of surgical patients collected in the years 2014–2019 to predict postoperative mortality using preoperatively available data. Models applied were Generalized Linear Model with regularization, Default Random Forest, Gradient Boosting Machine, eXtreme Gradient Boosting, Deep Learning and Stacked Ensembles comprising all base models. Further, we modified the original models by applying three different methods when training on the original pre-pandemic dataset: (1) we weighted older data weaker, (2) used only the most recent data for model training and (3) performed a z-transformation of the numerical input parameters. Afterwards, we tested model performance on a pre-pandemic and an in-pandemic data set not used in the training process, and analysed common features.

The second error was found in the legend of Fig. 4, following the abbreviation of ASA which mentions the number 34. It reads: ASA: American Society of Anaesthesiologists Physical Score 34.

The correct explanation should have referred to the source of Mayhew D, Mendonca V, Murthy BVS. A review of ASA physical status- historical perspectives and modern developments. Anaesthesia 2019;74:373–9. 10.1111/anae. 14,569. Thus, it should have read: ASA: American Society of Anaesthesiologists Physical Score [16].

Moreover, there is a typo with regards to the first line of the legend of Table 2 where the word “pandemic” was repeated twice. The correct legend should have read “The Kolmogorov-Smirnov-Test was performed between the pre-pandemic validation and the pre-pandemic test set”.

The original article [1] has been updated.
