# Supplementary material for: Susceptibility of AutoML mortality prediction algorithms to model drift caused by the COVID pandemic
Source: BMC Med Inform Decis Mak. 2024 Feb 2;24:34. doi: 10.1186/s12911-024-02428-z (PMC10837894; doi:10.1186/s12911-024-02428-z)
Supplement: Supplementary file 1 — Additional file 1. [file 12911_2024_2428_MOESM1_ESM.pdf]

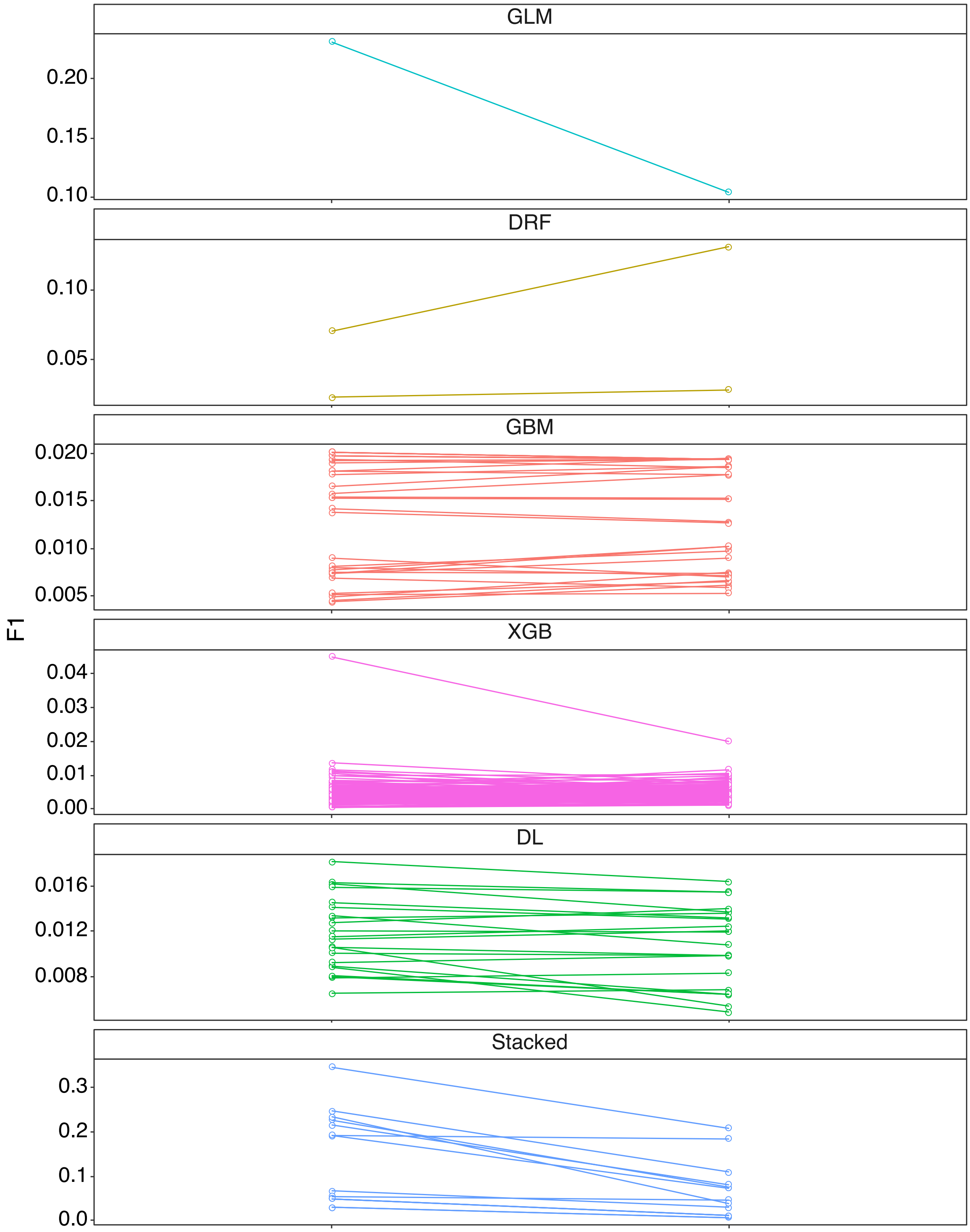


Supplemental Figure S1: F1-Scores of the native models on the pre-pandemic (left) and in-pandemic (right) test set. F1-scores represent the harmonic mean of precision and recall. Therefore, in-pandemic deterioration of F1-scores is present but more evident in the area under the precision-recall curve (Figure 2 in the main manuscript). It depends on clinical needs whether the F1-optimized threshold of the curve is suitable.

GLM: Generalized Linear Model, DRF: Default Random Forest, GBM: Gradient Boosting Machine, XGB: XGBoost, DL: Deep Learning, Stacked: Stacked Ensemble

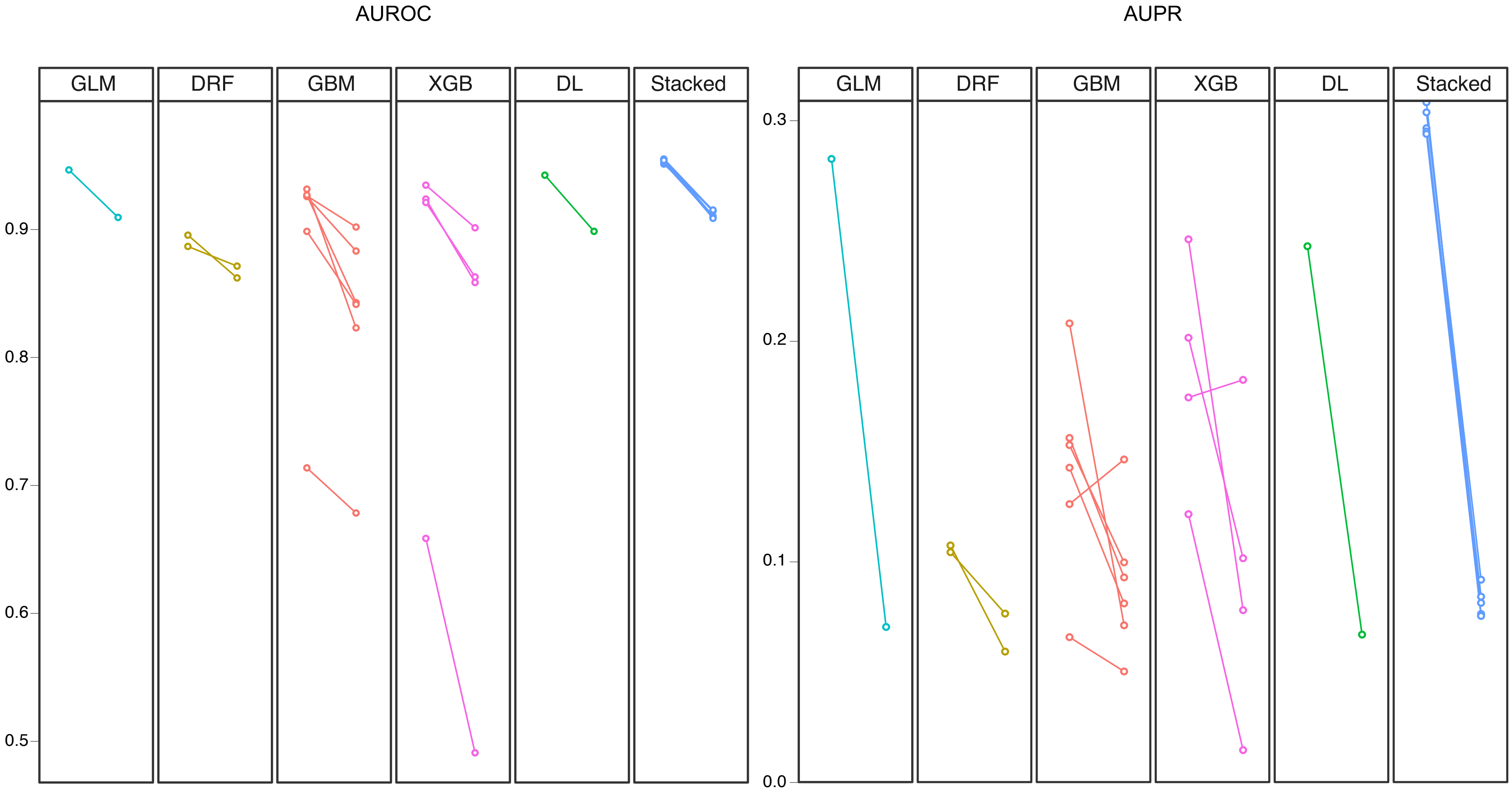


Supplemental Figure S2: Here, a z-transformation was performed on the pre-pandemic training/validation set and on the pre- and in-pandemic test set separately. The Figure depicts model performance on the pre-pandemic and in-pandemic test set on the right or left side of the respective box. Both AUROC and AUPR deteriorated during the pandemic for most models. AUROC: area under receiver-operating characteristics curve, AUPR: area under precision-recall curve, GLM: Generalized Linear Model, DRF: Default Random Forest, GBM: Gradient Boosting Machine, XGB: XGBoost, DL: Deep Learning, Stacked: Stacked Ensemble

**Table S1: Model performance**

**Weighted**

| | | AUROC | | | | | AUPRC | | | | |
|---|---|---|---|---|---|---|---|---|---|---|---|
| model | n | validation set | pre-pandemic | in-pandemic | % Change | p-value | validation set | pre-pandemic | in-pandemic | % Change | p-value |
| GLM | 1 | 0.92 | 0.93 | 0.90 | -3.95 | | 0.16 | 0.19 | 0.12 | -38.76 | |
| DRF | 2 | 0.90 (0.01) | 0.93 (0.01) | 0.87 (0.02) | -6.09 (1.96) | 0.14 | 0.13 (0.03) | 0.18 (0.05) | 0.06 (0.01) | -66.47 (4.05) | 0.15 |
| GBM | 12 | 0.89 (0.02) | 0.92 (0.03) | 0.85 (0.04) | -7.91 (4.47) | <0.001 | 0.11 (0.02) | 0.26 (0.06) | 0.08 (0.02) | -66.65 (12.65) | <0.001 |
| XGBoost | 29 | 0.90 (0.01) | 0.94 (0.01) | 0.89 (0.02) | -4.82 (1.36) | <0.001 | 0.12 (0.02) | 0.25 (0.05) | 0.07 (0.02) | -70.76 (8.25) | <0.001 |
| Deep Learning | 5 | 0.64 (0.09) | 0.61 (0.14) | 0.67 (0.08) | 12.30 (20.53) | 0.22 | 0.04 (0.02) | 0.04 (0.03) | 0.04 (0.02) | -9.28 (23.27) | 0.41 |
| Stacked | 13 | 0.95 (0.01) | 0.95 (0.01) | 0.91 (0.01) | -4.55 (0.61) | <0.001 | 0.27 (0.19) | 0.27 (0.03) | 0.10 (0.02) | -61.69 (14.71) | <0.001 |
| All | 62 | 0.89 (0.08) | 0.91 (0.10) | 0.87 (0.07) | -4.01 (7.59) | <0.001 | 0.14 (0.11) | 0.24 (0.07) | 0.08 (0.02) | -62.45 (20.40) | <0.001 |

**6 months**

| | | AUROC | | | | | AUPRC | | | | |
|---|---|---|---|---|---|---|---|---|---|---|---|
| model | n | validation set | pre-pandemic | in-pandemic | % Change | p-value | validation set | pre-pandemic | in-pandemic | % Change | p-value |
| GLM | 1 | 0.88 | 0.90 | 0.87 | -3.73 | | 0.08 | 0.11 | 0.07 | -37.26 | |
| DRF | 1 | 0.82 | 0.86 | 0.85 | -1.84 | | 0.04 | 0.08 | 0.05 | -34.95 | |
| GBM | 44 | 0.74 (0.10) | 0.76 (0.11) | 0.76 (0.11) | 0.23 (5.63) | 0.82 | 0.09 (0.03) | 0.08 (0.03) | 0.05 (0.02) | -28.60 (24.46) | <0.001 |
| XGBoost | 203 | 0.90 (0.02) | 0.92 (0.01) | 0.86 (0.02) | -6.22 (1.81) | <0.001 | 0.10 (0.03) | 0.17 (0.03) | 0.07 (0.01) | -59.54 (10.71) | <0.001 |
| Deep Learning | 9 | 0.80 (0.08) | 0.85 (0.05) | 0.81 (0.06) | -4.84 (3.86) | 0.005 | 0.08 (0.04) | 0.11 (0.02) | 0.06 (0.01) | -43.50 (18.93) | <0.001 |
| Stacked | 14 | 0.95 (0.03) | 0.93 (0.01) | 0.88 (0.01) | -5.57 (0.55) | <0.001 | 0.30 (0.29) | 0.16 (0.04) | 0.08 (0.01) | -49.42 (12.53) | <0.001 |
| All | 272 | 0.87 (0.08) | 0.89 (0.07) | 0.84 (0.06) | -5.07 (3.68) | <0.001 | 0.11 (0.08) | 0.15 (0.04) | 0.07 (0.01) | -53.32 (18.25) | <0.001 |

**Scaled**

| | | AUROC | | | | | AUPRC | | | | |
|---|---|---|---|---|---|---|---|---|---|---|---|
| model | n | validation set | pre-pandemic | in-pandemic | % Change | p-value | validation set | pre-pandemic | in-pandemic | % Change | p-value |
| GLM | 1 | 0.93 | 0.95 | 0.91 | -4.14 | | 0.17 | 0.27 | 0.06 | -76.22 | |
| DRF | 2 | 0.85 (0.01) | 0.90 (0.00) | 0.87 (0.00) | -3.22 (0.01) | 0.004 | 0.05 (0.00) | 0.13 (0.02) | 0.09 (0.03) | -30.42 (8.07) | 0.046 |
| GBM | 10 | 0.83 (0.04) | 0.87 (0.04) | 0.78 (0.03) | -10.60 (2.70) | <0.001 | 0.09 (0.01) | 0.14 (0.04) | 0.07 (0.02) | -48.11 (19.47) | <0.001 |
| XGBoost | 18 | 0.89 (0.02) | 0.93 (0.01) | 0.88 (0.02) | -5.40 (1.81) | <0.001 | 0.10 (0.02) | 0.22 (0.03) | 0.07 (0.02) | -65.17 (9.65) | <0.001 |
| Deep Learning | 5 | 0.91 (0.02) | 0.94 (0.01) | 0.89 (0.01) | -5.15 (1.00) | <0.001 | 0.12 (0.03) | 0.20 (0.03) | 0.06 (0.01) | -70.37 (1.67) | <0.001 |
| Stacked | 10 | 0.94 (0.01) | 0.95 (0.00) | 0.91 (0.02) | -4.89 (1.42) | <0.001 | 0.27 (0.18) | 0.28 (0.04) | 0.07 (0.01) | -74.75 (3.55) | <0.001 |
| All | 46 | 0.89 (0.05) | 0.92 (0.04) | 0.87 (0.05) | -6.27 (2.96) | <0.001 | 0.14 (0.11) | 0.21 (0.06) | 0.07 (0.02) | -62.84 (15.94) | <0.001 |

Model performance on the validation set and on the pre- and in-pandemic test sets of the "weighted", "6 months", and "scaled" method (in this table, "scaled" means that the entire dataset has been scaled). Pre- and in-pandemic AUROC and AUPRC are compared using paired t-test where $p<0.05$ was considered statistically significant. % Change indicates the mean percentage change of each model family. GLM: Generalized Linear Model, DRF: Default Random Forest, GBM: Gradient Boosting Machine,

## Table S2: pre- and in-pandemic evaluation metrics

values are given as mean (sd)

GLM: generalized linear model
DRF: distributed random forest
GBM: gradient boosting machine
Stacked: stacked ensemble
AUROC: area under receiver operating characteristic curve
AUPR: area under precision-recall curve

p-values: **< 0.05 bold** ≥ 0.05 gray
ANOVA p-value and post-hoc Tukey HSD for comparison of the methods "native", "weight", "six months" and "scaled", scaling performed on the whole dataset

| | | | native, n = 1 | weight, n = 1 | six months, n = 1 | scale, n = 1 |
|---|---|---|---|---|---|---|
| GLM | AUROC | pre pandemic | 0.94 (NA) | 0.93 (NA) | 0.90 (NA) | 0.95 (NA) |
| | | in pandemic | 0.90 (NA) | 0.90 (NA) | 0.87 (NA) | 0.91 (NA) |
| | | delta | -0.04 (NA) | -0.04 (NA) | -0.03 (NA) | -0.04 (NA) |
| | | percent | -3.85 (NA) | -3.95 (NA) | -3.73 (NA) | -4.14 (NA) |
| | AUPR | pre pandemic | 0.19 (NA) | 0.19 (NA) | 0.11 (NA) | 0.27 (NA) |
| | | in pandemic | 0.08 (NA) | 0.12 (NA) | 0.07 (NA) | 0.06 (NA) |
| | | delta | -0.11 (NA) | -0.07 (NA) | -0.04 (NA) | -0.20 (NA) |
| | | percent | -57.65 (NA) | -38.76 (NA) | -37.26 (NA) | -76.22 (NA) |

| | | | native, n = 2 | weight, n = 2 | six months, n = 1 | scale, n = 2 | p-value | native-weight | native-six months | native-scale | weight-six months | weight-scale | scale-six months |
|---|---|---|---|---|---|---|---|---|---|---|---|---|---|
| DRF | AUROC | pre pandemic | 0.90 (0.00) | 0.93 (0.01) | 0.86 (NA) | 0.90 (0.00) | 0.006 | 0.023 | 0.036 | 0.999 | 0.005 | 0.024 | 0.034 |
| | | in pandemic | 0.85 (0.02) | 0.87 (0.02) | 0.85 (NA) | 0.87 (0.00) | 0.6 | | | | | | |
| | | delta | -0.04 (0.01) | -0.06 (0.02) | -0.02 (NA) | -0.03 (0.00) | 0.2 | | | | | | |
| | | percent | -4.98 (1.59) | -6.09 (1.96) | -1.84 (NA) | -3.22 (0.01) | 0.2 | | | | | | |
| | AUPR | pre pandemic | 0.09 (0.02) | 0.18 (0.05) | 0.08 (NA) | 0.13 (0.02) | 0.2 | | | | | | |
| | | in pandemic | 0.07 (0.00) | 0.06 (0.01) | 0.05 (NA) | 0.09 (0.03) | 0.3 | | | | | | |
| | | delta | -0.03 (0.02) | -0.12 (0.04) | -0.03 (NA) | -0.04 (0.00) | 0.10 | | | | | | |
| | | percent | -26.26 (15.57) | -66.47 (4.05) | -34.95 (NA) | -30.42 (8.07) | 0.085 | | | | | | |

| | | | native, n = 32 | weight, n = 12 | six months, n = 44 | scale, n = 10 | p-value | native-weight | native-six months | native-scale | weight-six months | weight-scale | six months-scale |
|---|---|---|---|---|---|---|---|---|---|---|---|---|---|
| GBM | AUROC | pre pandemic | 0.86 (0.06) | 0.92 (0.03) | 0.76 (0.11) | 0.87 (0.04) | <0.001 | 0.168 | 0.000 | 0.999 | 0.000 | 0.418 | 0.002 |
| | | in pandemic | 0.81 (0.06) | 0.85 (0.04) | 0.76 (0.11) | 0.78 (0.03) | 0.009 | 0.524 | 0.095 | 0.665 | 0.014 | 0.179 | 0.982 |
| | | delta | -0.05 (0.04) | -0.07 (0.04) | 0.00 (0.04) | -0.09 (0.03) | <0.001 | 0.537 | 0.000 | 0.055 | 0.000 | 0.681 | 0.000 |
| | | percent | -6.26 (5.17) | -7.91 (4.47) | 0.23 (5.63) | -10.60 (2.70) | <0.001 | 0.778 | 0.000 | 0.097 | 0.000 | 0.612 | 0.000 |
| | AUPR | pre pandemic | 0.19 (0.04) | 0.26 (0.06) | 0.08 (0.03) | 0.14 (0.04) | <0.001 | 0.000 | 0.000 | 0.004 | 0.000 | 0.000 | 0.000 |
| | | in pandemic | 0.07 (0.03) | 0.08 (0.02) | 0.05 (0.02) | 0.07 (0.02) | <0.001 | 0.760 | 0.006 | 0.992 | 0.005 | 0.742 | 0.223 |
| | | delta | -0.12 (0.05) | -0.18 (0.06) | -0.03 (0.03) | -0.07 (0.04) | <0.001 | 0.001 | 0.000 | 0.029 | 0.000 | 0.000 | 0.014 |
| | | percent | -59.95 (18.33) | -66.65 (12.65) | -28.60 (24.46) | -48.11 (19.47) | <0.001 | 0.781 | 0.000 | 0.407 | 0.000 | 0.172 | 0.045 |

| | | | native, n = 198 | weight, n = 29 | six months, n = 203 | scale, n = 18 | p-value | native-weight | native-six months | native-scale | weight-six months | weight-scale | six months-scale |
|---|---|---|---|---|---|---|---|---|---|---|---|---|---|
| XGBoost | AUROC | pre pandemic | 0.93 (0.01) | 0.94 (0.01) | 0.92 (0.01) | 0.93 (0.01) | <0.001 | 0.150 | 0.000 | 0.950 | 0.000 | 0.772 | 0.000 |
| | | in pandemic | 0.88 (0.02) | 0.89 (0.02) | 0.86 (0.02) | 0.88 (0.02) | <0.001 | 0.040 | 0.000 | 0.995 | 0.000 | 0.400 | 0.000 |
| | | delta | -0.05 (0.02) | -0.05 (0.01) | -0.06 (0.02) | -0.05 (0.02) | <0.001 | 0.489 | 0.000 | 0.999 | 0.002 | 0.731 | 0.341 |
| | | percent | -5.36 (1.87) | -4.82 (1.36) | -6.22 (1.81) | -5.40 (1.81) | <0.001 | 0.451 | 0.000 | 1.000 | 0.001 | 0.718 | 0.247 |
| | AUPR | pre pandemic | 0.24 (0.04) | 0.25 (0.05) | 0.17 (0.03) | 0.22 (0.03) | <0.001 | 0.244 | 0.000 | 0.037 | 0.000 | 0.004 | 0.000 |
| | | in pandemic | 0.07 (0.01) | 0.07 (0.02) | 0.07 (0.01) | 0.07 (0.02) | 0.014 | 0.088 | 0.604 | 0.060 | 0.307 | 0.959 | 0.186 |
| | | delta | -0.17 (0.04) | -0.18 (0.05) | -0.10 (0.03) | -0.14 (0.04) | <0.001 | 0.830 | 0.000 | 0.003 | 0.000 | 0.003 | 0.000 |
| | | percent | -72.08 (8.10) | -70.76 (8.25) | -59.54 (10.71) | -65.17 (9.65) | <0.001 | 0.896 | 0.000 | 0.016 | 0.000 | 0.200 | 0.074 |

| | | | native, n = 22 | weight, n = 5 | six months, n = 9 | scale, n = 5 | p-value | native-weight | native-six months | native-scale | weight-six months | weight-scale | six months-scale |
|---|---|---|---|---|---|---|---|---|---|---|---|---|---|
| Deep Learning | AUROC | pre pandemic | 0.90 (0.02) | 0.61 (0.14) | 0.85 (0.05) | 0.94 (0.01) | <0.001 | 0.000 | 0.129 | 0.353 | 0.000 | 0.000 | 0.020 |
| | | in pandemic | 0.84 (0.04) | 0.67 (0.08) | 0.81 (0.06) | 0.89 (0.01) | <0.001 | 0.000 | 0.324 | 0.144 | 0.000 | 0.000 | 0.015 |
| | | delta | -0.06 (0.03) | 0.06 (0.09) | -0.04 (0.03) | -0.05 (0.01) | <0.001 | 0.000 | 0.762 | 0.977 | 0.000 | 0.001 | 0.987 |
| | | percent | -6.30 (3.15) | 12.29 (20.52) | -4.84 (3.86) | -5.15 (1.00) | <0.001 | 0.000 | 0.959 | 0.989 | 0.001 | 0.003 | 1.000 |
| | AUPR | pre pandemic | 0.14 (0.03) | 0.04 (0.03) | 0.11 (0.02) | 0.20 (0.03) | <0.001 | 0.000 | 0.022 | 0.000 | 0.001 | 0.000 | 0.000 |
| | | in pandemic | 0.05 (0.01) | 0.04 (0.02) | 0.06 (0.02) | 0.06 (0.01) | 0.011 | 0.111 | 0.349 | 0.507 | 0.012 | 0.029 | 1.000 |
| | | delta | -0.09 (0.03) | -0.01 (0.02) | -0.05 (0.03) | -0.14 (0.02) | <0.001 | 0.000 | 0.001 | 0.000 | 0.038 | 0.000 | 0.000 |
| | | percent | -62.17 (7.57) | -9.27 (23.28) | -41.79 (22.33) | -70.37 (1.67) | <0.001 | 0.000 | 0.004 | 0.647 | 0.001 | 0.000 | 0.005 |

| | | | native, n = 14 | weight, n = 13 | six months, n = 14 | scale, n = 10 | p-value | native-weight | native-six months | native-scale | weight-six months | weight-scale | six months-scale |
|---|---|---|---|---|---|---|---|---|---|---|---|---|---|
| Stacked | AUROC | pre pandemic | 0.95 (0.01) | 0.95 (0.01) | 0.93 (0.01) | 0.95 (0.00) | <0.001 | 0.956 | 0.000 | 1.000 | 0.000 | 0.960 | 0.000 |
| | | in pandemic | 0.91 (0.01) | 0.91 (0.01) | 0.88 (0.01) | 0.91 (0.02) | <0.001 | 0.873 | 0.000 | 0.974 | 0.000 | 0.683 | 0.000 |
| | | delta | -0.04 (0.01) | -0.04 (0.01) | -0.05 (0.01) | -0.05 (0.01) | 0.044 | 0.966 | 0.109 | 0.955 | 0.042 | 0.789 | 0.389 |
| | | percent | -4.70 (0.73) | -4.55 (0.61) | -5.57 (0.55) | -4.89 (1.42) | 0.014 | 0.965 | 0.042 | 0.950 | 0.014 | 0.775 | 0.217 |
| | AUPR | pre pandemic | 0.26 (0.03) | 0.27 (0.03) | 0.16 (0.04) | 0.28 (0.04) | <0.001 | 0.957 | 0.000 | 0.476 | 0.000 | 0.772 | 0.000 |
| | | in pandemic | 0.09 (0.00) | 0.10 (0.02) | 0.08 (0.01) | 0.07 (0.01) | <0.001 | 0.129 | 0.411 | 0.022 | 0.003 | 0.000 | 0.404 |
| | | delta | -0.17 (0.03) | -0.17 (0.05) | -0.08 (0.03) | -0.21 (0.04) | <0.001 | 0.990 | 0.000 | 0.107 | 0.000 | 0.063 | 0.000 |
| | | percent | -65.91 (6.86) | -61.69 (14.71) | -49.42 (12.53) | -74.75 (3.55) | <0.001 | 0.736 | 0.001 | 0.203 | 0.023 | 0.027 | 0.000 |

**Table S3: Top 10 most important parameters in the best model of each family**

| | native | | | | weight | | | | scaled | | | | six months | | | |
|---|---|---|---|---|---|---|---|---|---|---|---|---|---|---|---|---|
| | pre pandemic | | in pandemic | | pre pandemic | | in pandemic | | pre pandemic | | in pandemic | | pre pandemic | | in pandemic | |
| | parameter | scaled importance | parameter | scaled importance | parameter | scaled importance | parameter | scaled importance | parameter | scaled importance | parameter | scaled importance | parameter | scaled importance | parameter | scaled importance |
| **GLM** | age | 1,00 | age | 1,00 | age | 1,00 | age | 1,00 | age | 1,00 | age | 1,00 | OPS: 5.010.10 | 1,00 | OPS: 5.010.10 | 1,00 |
| | no of ordered RPC's | 0,66 | no of ordered RPC's | 0,66 | no of ordered RPC's | 0,75 | no of ordered RPC's | 0,75 | department of surgery: neurosurgery | 0,49 | department of surgery: neurosurgery | 0,49 | age | 0,96 | age | 0,96 |
| | no of preop consults | 0,56 | no of preop consults | 0,56 | no of preop consults | 0,59 | no of preop consults | 0,59 | department of surgery: surgery | 0,40 | department of surgery: surgery | 0,40 | OPS: 5.541.0 | 0,95 | OPS: 5.541.0 | 0,95 |
| | OPS: 5.010.10 | 0,47 | OPS: 5.010.10 | 0,47 | OPS: 5.010.10 | 0,51 | OPS: 5.010.10 | 0,51 | surgery within regular service | 0,35 | surgery within regular service | 0,35 | OPS: 5.541.1 | 0,83 | OPS: 5.541.1 | 0,83 |
| | OPS: 5.022.00 | 0,45 | OPS: 5.022.00 | 0,45 | OPS: 5.022.00 | 0,49 | OPS: 5.022.00 | 0,49 | OPS: 5.469.20 | 0,34 | OPS: 5.469.20 | 0,34 | OPS: 5.022.00 | 0,82 | OPS: 5.022.00 | 0,82 |
| | reason for admission | 0,40 | reason for admission | 0,40 | international normalized ratio | 0,43 | international normalized ratio | 0,43 | OPS: 5.010.2 | 0,31 | OPS: 5.010.2 | 0,31 | OPS: 5.011.2 | 0,80 | OPS: 5.011.2 | 0,80 |
| | c-reactive protein | 0,37 | c-reactive protein | 0,37 | c-reactive protein | 0,41 | c-reactive protein | 0,41 | admission from external hospital | 0,30 | admission from external hospital | 0,30 | OPS: 5.829.c | 0,80 | OPS: 5.829.c | 0,80 |
| | international normalized ratio | 0,36 | international normalized ratio | 0,36 | reason for admission | 0,40 | reason for admission | 0,40 | planned admission | 0,29 | planned admission | 0,29 | urine trichomonads present | 0,78 | urine trichomonads present | 0,78 |
| | days of prehospital treatment | 0,35 | days of prehospital treatment | 0,35 | blood urea nitrogen | 0,36 | blood urea nitrogen | 0,36 | department of surgery: ophthalmology | 0,29 | department of surgery: ophthalmology | 0,29 | OPS: 5.454.20 | 0,76 | OPS: 5.454.20 | 0,76 |
| | OPS: 5.013.1 | 0,34 | OPS: 5.013.1 | 0,34 | OPS: 5.013.1 | 0,36 | OPS: 5.013.1 | 0,36 | OPS: 5.022.00 | 0,27 | OPS: 5.022.00 | 0,27 | procalcitonine available | 0,75 | procalcitonine available | 0,75 |
| **DRF** | age | 1,00 | age | 1,00 | no of ordered RPC's | 1,00 | no of ordered RPC's | 1,00 | department of surgery | 1,00 | department of surgery | 1,00 | allergy to metamizol | 1,00 | allergy to metamizol | 1,00 |
| | location of premedication | 0,96 | location of premedication | 0,96 | albumin | 0,87 | albumin | 0,87 | lower jaw full denture | 0,86 | lower jaw full denture | 0,86 | ATC: S01A | 0,94 | ATC: S01A | 0,94 |
| | allergy to penicillin | 0,64 | allergy to penicillin | 0,64 | no of preop consults | 0,82 | no of preop consults | 0,82 | reason for admission | 0,80 | reason for admission | 0,80 | location of premedication | 0,57 | location of premedication | 0,57 |
| | location of premedication available | 0,60 | location of premedication available | 0,60 | haemoglobin | 0,78 | haemoglobin | 0,78 | age | 0,80 | age | 0,80 | allergy to latex | 0,54 | allergy to latex | 0,54 |
| | lower jaw partial denture | 0,57 | lower jaw partial denture | 0,57 | platelet count | 0,78 | platelet count | 0,78 | allergy to diclofenac | 0,58 | allergy to diclofenac | 0,58 | age | 0,52 | age | 0,52 |
| | difficult spinal puncture in history | 0,55 | difficult spinal puncture in history | 0,55 | haematocrit (lab) | 0,58 | haematocrit (lab) | 0,58 | past anaesthesia without findings | 0,54 | past anaesthesia without findings | 0,54 | past anaesthesia with difficult intubation | 0,46 | past anaesthesia with difficult intubation | 0,46 |
| | reason for admission | 0,51 | reason for admission | 0,51 | blood urea nitrogen | 0,57 | blood urea nitrogen | 0,57 | haematocrit | 0,54 | haematocrit | 0,54 | reason for admission | 0,35 | reason for admission | 0,35 |
| | no of ordered RPC's | 0,49 | no of ordered RPC's | 0,49 | erythrocyte count | 0,56 | erythrocyte count | 0,56 | past anaesthesia with PONV | 0,54 | past anaesthesia with PONV | 0,54 | A12A | 0,30 | ATC: A12A | 0,30 |
| | hay fever | 0,44 | hay fever | 0,44 | age | 0,55 | age | 0,55 | past anaesthesia sore throat | 0,53 | past anaesthesia sore throat | 0,53 | allergy to plaster | 0,26 | allergy to plaster | 0,26 |
| | haematocrit | 0,42 | haematocrit | 0,42 | haematocrit POC | 0,53 | haematocrit POC | 0,53 | type of admission | 0,48 | type of admission | 0,48 | allergy to iodine | 0,26 | allergy to iodine | 0,26 |
| **GBM** | age | 1,00 | reason for admission | 1,00 | age | 1,00 | age | 1,00 | reason for admission | 1,00 | reason for admission | 1,00 | reason for admission | 1,00 | reason for admission | 1,00 |
| | ASA | 0,88 | location of premedication | 0,84 | no of ordered RPC's | 0,75 | albumin | 0,84 | ASA | 0,84 | department of surgery | 0,90 | days of prehospital treatment | 0,81 | days of prehospital treatment | 0,81 |
| | no of ordered FFP's | 0,60 | age | 0,65 | no of preop consults | 0,71 | no of ordered RPC's | 0,61 | time of surgery start | 0,46 | ASA | 0,83 | age | 0,79 | age | 0,79 |
| | location of premedication | 0,44 | ASA | 0,46 | time of surgery start | 0,62 | c-reactive protein | 0,50 | department of surgery | 0,44 | age | 0,56 | allergy to metamizol | 0,21 | allergy to metamizol | 0,21 |
| | reason for admission | 0,41 | location of premedication available | 0,26 | haemoglobin POC | 0,55 | no of preop consults | 0,49 | age | 0,39 | time of surgery start | 0,33 | ATC: A02B | 0,18 | ATC: A02B | 0,18 |
| | location of premedication available | 0,34 | c-reactive protein | 0,24 | haemoglobin (lab) | 0,47 | haemoglobin | 0,44 | no of ordered FFP's | 0,35 | c-reactive protein | 0,29 | mean corpuscular haemoglobin | 0,12 | mean corpuscular haemoglobin | 0,12 |
| | department of surgery | 0,33 | haemoglobin | 0,22 | albumin | 0,47 | time of surgery start | 0,44 | month of surgery | 0,33 | no of ordered FFP's | 0,27 | body mass index | 0,12 | body mass index | 0,12 |
| | no of preop consults | 0,29 | no of ordered FFP's | 0,18 | department of surgery | 0,45 | month of surgery | 0,42 | haemoglobin | 0,24 | no of ordered RPC's | 0,24 | location of premedication | 0,12 | location of premedication | 0,12 |
| | c-reactive protein | 0,21 | no of ordered RPC's | 0,15 | c-reactive protein | 0,45 | haematocrit | 0,38 | no of ordered RPC's | 0,14 | month of surgery | 0,24 | OPS: 5.011.2 | 0,10 | OPS: 5.011.2 | 0,10 |
| | days of prehospital treatment | 0,20 | no of preop consults | 0,14 | month of surgery | 0,37 | platelet count | 0,35 | c-reactive protein | 0,12 | haemoglobin | 0,19 | international normalized ratio | 0,10 | international normalized ratio | 0,10 |
| **XGBoost** | age | 1,00 | age | 1,00 | age | 1,00 | age | 1,00 | age | 1,00 | age | 1,00 | age | 1,00 | reason for admission | 1,00 |
| | no of preop consults | 0,72 | ASA | 0,87 | c-reactive protein | 0,79 | c-reactive protein | 0,70 | no of preop consults | 0,54 | location of premedication | 0,52 | body mass index | 0,55 | age | 0,43 |
| | c-reactive protein | 0,51 | reason for admission | 0,53 | no of ordered RPC's | 0,52 | no of ordered RPC's | 0,68 | c-reactive protein | 0,48 | haemoglobin | 0,38 | reason for admission | 0,51 | ATC: A02B | 0,18 |
| | time of surgery start | 0,45 | no of ordered FFP's | 0,37 | no of preop consults | 0,48 | no of preop consults | 0,62 | no of ordered RPC's | 0,40 | c-reactive protein | 0,31 | no of ordered RPC's | 0,37 | OPS: 5.011.2 | 0,17 |
| | leukocyte count | 0,41 | no of ordered RPC's | 0,34 | leukocyte count | 0,40 | troponin T | 0,33 | platelet count | 0,37 | no of ordered FFP's | 0,27 | troponin T | 0,34 | body mass index | 0,16 |
| | platelet count | 0,37 | haemoglobin | 0,32 | ASA | 0,38 | albumin | 0,31 | leukocyte count | 0,35 | no of preop consults | 0,22 | month of surgery | 0,28 | haemoglobin | 0,14 |
| | no of ordered RPC's | 0,37 | no of preop consults | 0,30 | body mass index | 0,36 | leukocyte count | 0,30 | body mass index | 0,23 | no of ordered RPC's | 0,22 | c-reactive protein | 0,27 | no of ordered RPC's | 0,11 |
| | department of surgery | 0,36 | c-reactive protein | 0,27 | platelet count | 0,32 | platelet count | 0,27 | gamma-glutamyl transferase | 0,23 | body mass index | 0,15 | weekday of surgery | 0,26 | type of admission | 0,10 |
| | body mass index | 0,32 | department of surgery | 0,21 | time of surgery start | 0,29 | body mass index | 0,26 | haematocrit | 0,23 | sodium | 0,14 | no of preop consults | 0,25 | international normalized ratio | 0,10 |
| | month of surgery | 0,30 | haematocrit | 0,18 | albumin | 0,28 | haemoglobin | 0,24 | blood urea nitrogen | 0,19 | ATC: R01B | 0,14 | mean corpuscular haemoglobin | 0,25 | month of surgery | 0,08 |
| **Deep Learning** | age | 1,00 | preop consults: radiology, emergency | 1,00 | age | 1,00 | age | 1,00 | age | 1,00 | age | 1,00 | preop consults: radiology, planned | 1,00 | time of surgery start | 1,00 |
| | OPS: 5.154.0 | 0,76 | preop consults: radiology, planned | 0,83 | monocyte count POC | 0,96 | monocyte count POC | 0,96 | c-reactive protein | 0,77 | cholesterolaemia | 0,72 | age | 0,87 | preop consults: radiology, planned | 0,96 |
| | OPS: 5.749.11 | 0,76 | age | 0,80 | Le-a antibody available | 0,96 | Le-a antibody available | 0,96 | preop consults: radiology, emergency | 0,67 | department of surgery | 0,71 | international normalized ratio | 0,78 | international normalized ratio | 0,95 |
| | ASA | 0,75 | c-reactive protein | 0,71 | GOT | 0,96 | GOT | 0,96 | Rh formula | 0,65 | time of surgery start | 0,58 | no of OPS code 5... | 0,76 | age | 0,91 |
| | OPS: 5.159.4 | 0,72 | international normalized ratio | 0,69 | lactate dehydrogenase determined | 0,96 | lactate dehydrogenase determined | 0,96 | albumin | 0,65 | remaining teeth | 0,57 | month of surgery | 0,74 | month of surgery | 0,82 |
| | OPS: 5.573.41 | 0,72 | OPS: 5.022.00 | 0,67 | cortisol | 0,95 | cortisol | 0,95 | no of ordered RPC's | 0,65 | OPS: 5.010.10 | 0,47 | sex | 0,66 | weekday of surgery | 0,75 |
| | OPS: 5.511.11 | 0,70 | no of preop consults | 0,57 | antibodies against ribonucleoprotein | 0,95 | antibodies against ribonucleoprotein | 0,95 | international normalized ratio | 0,61 | no of ordered RPC's | 0,46 | OPS: 5.011.2 | 0,65 | sex | 0,71 |
| | OPS: 5.144.5a | 0,70 | OPS: 8... present | 0,55 | kell result available | 0,95 | kell result available | 0,95 | no of preop consults | 0,60 | OPS: 5.159.4 | 0,46 | reason for admission | 0,61 | reason for admission | 0,70 |
| | OPS: 5.154.2 | 0,70 | OPS: 5.013.1 | 0,54 | mean corpuscular volume POC | 0,95 | mean corpuscular volume POC | 0,95 | monocyte count | 0,59 | reason for admission | 0,44 | department of surgery | 0,59 | preop consults: radiology, emergency | 0,68 |
| | OPS: 5.813.4 | 0,69 | sex | 0,53 | serum amyloid | 0,95 | serum amyloid | 0,95 | sodium | 0,59 | days of prehospital treatment | 0,44 | potassium | 0,56 | no of OPS code 5... | 0,64 |
| **Stacked** | age | 1,00 | pH | 1,00 | age | 1,00 | sodium | 1,00 | age | 1,00 | age | 1,00 | age | 1,00 | allergy to metamizol | 1,00 |
| | no of ordered RPC's | 0,48 | platelet count | 0,98 | c-reactive protein | 0,43 | GOT | 0,95 | no of ordered RPC's | 0,53 | no of ordered RPC's | 0,53 | reason for admission | 0,42 | allergy to latex | 1,00 |
| | c-reactive protein | 0,33 | blood group rapid test | 0,88 | no of ordered RPC's | 0,33 | age | 0,81 | no of ordered FFP's | 0,51 | no of ordered FFP's | 0,51 | body mass index | 0,29 | sodium | 0,91 |
| | no of preop consults | 0,30 | creatinine | 0,85 | no of preop consults | 0,32 | weekday of surgery | 0,81 | no of preop consults | 0,50 | no of preop consults | 0,50 | month of surgery | 0,24 | thyroid-stimulating hormone | 0,86 |
| | department of surgery | 0,28 | time of surgery start | 0,81 | platelet count | 0,29 | potassium | 0,76 | international normalized ratio | 0,50 | international normalized ratio | 0,50 | no of preop consults | 0,24 | ASA | 0,86 |
| | ASA | 0,26 | erythrocyte count | 0,79 | department of surgery | 0,28 | department of surgery | 0,60 | albumin | 0,49 | albumin | 0,49 | leukocyte count | 0,23 | mean corpuscular haemoglobin | 0,86 |
| | leukocyte count | 0,25 | MCH | 0,75 | body mass index | 0,27 | haemoglobin | 0,58 | c-reactive protein | 0,49 | c-reactive protein | 0,49 | time of surgery start | 0,21 | activated partial thromboplastin time | 0,83 |
| | body mass index | 0,19 | no of OPS code 5... | 0,72 | leukocyte count | 0,25 | mean corpuscular haemoglobin | 0,58 | sodium | 0,49 | sodium | 0,49 | type of admission | 0,19 | full denture | 0,80 |
| | reason for admission | 0,18 | thyroid-stimulating hormone | 0,63 | ASA | 0,25 | glutamate pyruvate transaminase | 0,55 | FFP's ordered | 0,48 | FFP's ordered | 0,48 | department of surgery | 0,19 | allergy to plaster | 0,78 |
| | haemoglobin | 0,17 | activated partial thromboplastin time | 0,63 | time of surgery start | 0,23 | erythrocyte count | 0,54 | MCH | 0,48 | MCH | 0,48 | no of ordered RPC's | 0,18 | no known allergy | 0,72 |

ASA: American society of anesthesiologists physical score, RPC's: red packed cells, FFP's fresh frozen plasma, GOT: glutamate oxaloacetate transaminase, POC: point of care testing, PONV: post-operative nausea and vomiting

ATC anatomic therapeutic chemical code:
A02B: Drugs for peptic ulcer and gastro-oesophageal reflux disease, A12A: Calcium, S01A: antiinfectives for ophthalmologic use, R01B: nasal decongestants for systemic use

OPS: surgical codes according to the German catalogue:
5.010.10 craniectomy, 5.010.2 drill hole trepanation, 5.011.2 transsphenoidal access, 5.013.1 Drainage of subdural haematoma, 5.022.00 extraventricular drainage, 5.144.5a phacoemulsification of the lens, 5.154.0 cryopexy, 5.154.2 laser retinopexy, 5.159.4 vitrectomy,
5.454.20 segmental resection of ileum, 5.469.20 adhesiolysis of the intestine, 5.511.11 laparoscopic cholecystectomy, 5.541.0 laparotomy, 5.541.1 laparotomy with drainage, 5.573.41 transurethral resection of the urinary bladder, 5.749.11 caesarean section,
5.813.4 cruciate ligament reconstruction, 5.829.c implantation or replacement of tumour endoprostheses,
8... Non-surgical therapeutic measures
